# Molecular diagnosis and biochemical studies of tick-borne diseases (anaplasmosis and babesiosis) in Aberdeen Angus Cattle in New Valley, Egypt

**DOI:** 10.14202/vetworld.2020.1884-1891

**Published:** 2020-09-16

**Authors:** Nani Nasreldin, Rania M. Ewida, Hatem Hamdon, Yasser F. Elnaker

**Affiliations:** 1 Department of Pathology and Clinical Pathology, Faculty of Veterinary Medicine, New Valley University, El-Kharga, P.O. Box 72511, Egypt; 2 Department of Food Hygiene (Milk Hygiene), Faculty of Veterinary Medicine, New Valley University, El-Kharga, P.O. Box 72511, Egypt; 3 Department of Animal Production, Faculty of Agriculture, New Valley University, El-Kharga, P.O. Box 72511, Egypt; 4 Department of Animal Medicines (Infectious Diseases), Faculty of Veterinary Medicine, New Valley University, El-Kharga, P.O. Box 72511, Egypt

**Keywords:** Aberdeen Angus cattle, *Anaplasma marginal*, *Babesia bovis*, oxidative stress

## Abstract

**Background and Aim:**

Anaplasmosis and babesiosis are tick-borne diseases that threaten livestock production with subsequent considerable economic losses. This study was conducted to diagnose *Anaplasma* and *Babesia* infection using molecular techniques in imported Aberdeen Angus cattle imported from Uruguay to El-Kharga Oasis in New Valley, Egypt, and to investigate the effects of disease on some serum biochemical and oxidative stress parameters.

**Materials and Methods:**

Blood samples were collected from 31 cattle, 21 diseased and ten apparently normal, of varying ages and sex. The blood was used for the preparation of blood smears, polymerase chain reaction assay, and separation of serum for biochemical investigation. The experimental production farm at the Faculty of Agriculture, New Valley University, was infested with ticks and variable clinical manifestations during the period from December 2017 to March 2018. One calf died of a suspected blood parasite infection.

**Results:**

The blood film examination revealed infection by blood parasites in 21 samples. *Anaplasma marginale* and *Babesia bovis* were identified in 12 and 14 samples, respectively. A total of 14 samples were examined by polymerase chain reaction (PCR) to make these identifications. Biochemical parameters showed significantly elevated serum alanine aminotransferase, aspartate aminotransferase, total bilirubin (T. Bil), and urea in blood from parasite-infected female cattle and male calves compared with controls. Increased serum total protein, globulin, and creatinine were recorded only in infected female cattle. The blood glucose level was significantly decreased in infected female cattle and male calves compared with controls. Furthermore, albumin and albumin/globulin ratio was significantly reduced in the infected female cattle. Oxidative stress profiles of infected animals showed a significant increase in serum nitric oxide and malondialdehyde, and both total antioxidant capacity and reduced glutathione (GSH) were significantly reduced in comparison with control animals.

**Conclusion:**

The incidence of *A. marginale* and *B. bovis* infection is high in imported Aberdeen Angus cattle in New Valley Province. PCR methods provide a short-term assessment of disease. An extensive epidemiological survey, employing serology together with molecular genetic methods, monitoring of abundance and distribution of tick vectors, availability of vaccination programs, and tracking of animal transport is also needed for control of blood parasites.

## Introduction

Bovine anaplasmosis and babesiosis are tick-borne diseases that cause significant economic losses in many countries. Bovine anaplasmosis is regarded as economically important infectious non-contagious rickettsial disease that affect ruminants. In recovered animals, the epizootiology of *Anaplasma* infection is complicated by a life-long carrier state [1].

Anaplasmosis is caused by *Anaplasma marginale*, *A. phagocytophilum*, *A. centrale*, and *A. bovis* [2]. *A. marginale*, which is primarily the etiologic agent of bovine anaplasmosis, is widespread globally and endemic in some regions in Africa and Asia [3,4]. The prevalence of *Anplasma* infection differs by locality, endemic tick species, bioclimate, animal breed, and type of breeding [1]. Bovine anaplasmosis in Egypt is a significant constraint to livestock improvement programs. The disease causes serious health problems leading to substantial economic losses and reductions in animal productivity [5]. *A. marginale* is transmitted biologically by ticks or mechanically through blood-contaminated fomites, mouthparts of biting flies, horseflies, bloodsucking flies, and mosquitoes [4,6]. Mature erythrocytes are infected by *A. marginale* through endocytosis then released by exocytosis to attack other erythrocytes. The infection leads to a drastic increase in the number of infected erythrocytes. These cells are then phagocytosed by reticuloendothelial cells leading to hemolytic anemia and icterus [7]. Clinical signs of *A. marginale* infection include anorexia, dyspnea, tachycardia, hemolytic anemia, jaundice, lacrimation, salivation, fever, fatigue, diarrhea, frequent urination, and abortion. In some cases, the infection leads to death of the animal in less than one day [8]. Bovine babesiosis is an important tick-borne disease worldwide, but mainly in tropical and subtropical regions. In Egypt, the disease is caused by *Babesia bovis* and *B. bigemina* [9]. *B. bovis* is an apicomplexan protozoon that parasitizes red blood cells. The protozoon is transmitted from animal to animal by cattle ticks (transovarial transmission), and it differs in lifecycle from *Anaplasma* spp. Clinical signs of *B. bovis* infection are depression, fever, anemia, hemoglobinuria, jaundice, diarrhea, and abortion. Muscle wasting, tremor, convulsions, and coma also commonly occur. Neurological signs include circling, aggression, nystagmus, hyperesthesia convulsions, and paralysis [10]. Babesiosis and anaplasmosis are separate diseases that often co-exist in the same animal [4]. In general, oxidative stress is defined as an imbalance between oxidants and antioxidant levels. Such stress may occur when antioxidant enzymes are reduced or other conditions where uncontrolled production of free radicals arises. Unregulated generation of free radicals causes significant damage to cellular structures. Malondialdehyde (MDA) is an end product of lipid peroxidation, resulting from reactions between reactive oxygen species (ROS) and polyunsaturated fatty acids in the cell membrane. MDA is used as a biomarker for free radical-mediated damage. Nitric oxide (NO) also modulates host defense mechanisms against numerous intracellular parasitic diseases. Changes in indices of oxidative stress are recorded in parasitic diseases. The role of erythrocytic peroxidation in the pathogenesis of several hemiparasitic infections is well recognized [7,11].

This study was conducted to diagnose *Babesia* and *Anaplasma* infection in Aberdeen Angus cattle in New Valley, Egypt, using molecular methods. Further, the study examined the effects of parasitic infection on some serum biochemical and oxidative stress parameters.

## Materials and Methods

### Ethical approval

This study was carried out according to the regulation and procedures approved by the ethics committee on animal experimentation of the New Valley University, Faculty of Veterinary Medicine and the guide for the care and use of animals (National Institute of Health Publication NO. 8023, revised 1978)

### Study period and study area

The study was performed in the period extending from December 2017 to March 2018 in El-Kharga city, New Valley governorate, Egypt. El-Kharga Oasis occupies a depression in the southern part of the western desert. It lies on 25.4390 ° N latitude and 30.5586 ° E longitude (25°26 ′ 56″ N and 30°32′ 24″ E). It is located southwest of Egypt and is situated 600 Km to the south of Cairo governorate. The climate of this region is arid and dry, basically that of the desert. Rainfall is nearly negligible and the ambient temperature ranges from 46°C through the summer days to 8°C at chilly winter nights.

### Study animals

The study used 40 Aberdeen Angus cattle. Animals included 27 females, 21 aged 3-4 years, and 6 older than 4 years. Thirteen male animals were also included, two aged 1.6 years, and 11 <1 year old. All animals were kept at the Animal Production Experimental Farm of the Faculty of Agriculture, The New Valley University. This herd was introduced to the farm in 2014. Animals were imported from Uruguay through the Egyptian Ministry of Agriculture. All animals were fed on a basal diet formulated for beef cattle [12]. The basal diet consisted of 40% wheat straw and 60% concentrate. Water was supplied *ad libitum*. Animals were dewormed and regularly vaccinated following the Egyptian Authority Program.

### Sampling

#### Blood samples

Thirty-one blood samples (21 diseased and ten clinically normal animals) were collected from the jugular vein into tubes with EDTA for preparation of blood film. Fourteen blood samples were used for PCR assay.

#### Serum samples

Thirty-one blood samples were collected into plain tubes for serum separation. Tubes were kept in an inclined position for 20 min at room temperature (20-25°C). The samples were then refrigerated to inhibit glycolysis and for complete retraction of the blood clot. Subsequently, the samples were centrifuged at 3000 rpm for 10 min to separate clear serum. Serum was stored in Eppendorf tubes at −80°C until use in biochemical analyses.

### Preparation of blood smear

Blood films were made manually as soon as possible after the collection of blood samples. Two blood films were made per blood sample. Films were stained with Giemsa [13]. Blood smears were prepared and examined under an oil immersion lens in the laboratory of Faculty of Veterinary Medicine, New Valley University.

### PCR

PCR used DNA from 14 blood samples and was carried out at the Molecular Biology Research Institute, Assiut University (ISO/IEC 17025:2017). Primers specific for *B. bovis* and *A. marginale* were used.

#### DNA extraction of blood samples

DNA was extracted from whole blood using a Qiagen DNA Blood Mini Kit (Cat. No. 51104, Helden, Germany) following the manufacturer’s instructions.

#### PCR amplification primers

The MAR1bB2 primer set was used for *A. marginale–* Forward: 50- GCT CTA GCA GGT TAT GCG TC-30; Reverse: 50-CTG CTT GGG AGA ATG CAC CT-30). Primers were designed to specifically amplify a 265 bp region of the major surface protein–1b encoding gene.

The bovar2A primer set was used for *B. bovis –* Forward: 50-CAA GCA TAC AAC CAG GTG G–30; Reverse: 50-ACCCCA GGC ACA TCC AGC TA–30). Primers were designed to amplify a 166 bp region of the multi-copy *VESA–1a* gene.

Each set of primer pairs was checked for specificity using the BLASTN algorithm in conjunction with the NCBI database (http://blast.ncbi.nlm.nih.gov/Blast.cgi). For PCR reaction, a total volume of 25 μL (12.5 μL of 2× PCR master mix [Green Master, Promega, USA], 150 ng of DNA template, 1 μL of each primer and nuclease-free water to 25 μL). PCR assays were performed using a Gradient Thermal Cycler (Veriti Applied Biosystem, USA) under the following conditions: After an initial denaturation at 96°C for 60 s, 35 cycles followed by denaturation at 96°C for 15 s, annealing at 60°C for 1 min, extension at 72°C for 30 s, and a final extension step at 72°C for 10 min.

### Gel electrophoresis

PCR products were separated on 1% agarose gels (GX 040.90 Gen Agarose, L.E., Standard DNA/RNA agarose Molecular Biology Grade, Inno-Train Diagnostic, D-61476, Kronberg/Taunus) containing ethidium bromide in 1 μL/mL electrophoresis buffer at 100 volt for 60 min. A 100 bp DNA-Ladder was used to quantify MW (SciE-PLAS, HU10, 5636, UK). The results were visualized with a high-performance ultraviolet transilluminator (Uv, JNC, UK). Images of PCR products containing the DNA sequence of target genes were evaluated using Doc. It^®^ LS, Image acquisition software (UVP, JNC, UK).

### Serum biochemical analysis

Alanine aminotransferase (ALT), aspartate aminotransferase (AST), total protein (TP), albumin, urea, and creatinine were analyzed using commercial kits (Human Co. Germany). Globulin concentration and albumin/globulin (A/G) ratio was calculated as previously described [14]. For oxidative stress studies, serum MDA, NO, GSH, and total antioxidant capacity (TAC) were determined with commercial kits (Biodiagnostic, Egypt). All serum biochemical and oxidative stress parameters were assayed spectrophotometrically (5010 V5+, photometer, RIELE Co. Germany) following the manufacturer’s instructions.

### Statistical analysis

Serum biochemical and oxidative stress ­parameters were statistically analyzed with independent-sample t-tests using program SPSS software (version 23.0) for Windows (IBM Corp. Armonk, NY, USA). The results are presented as mean ± SE and p<0.05 was assumed to reflect statistical significance.

## Results

### Clinical examination

**Figure-1:**
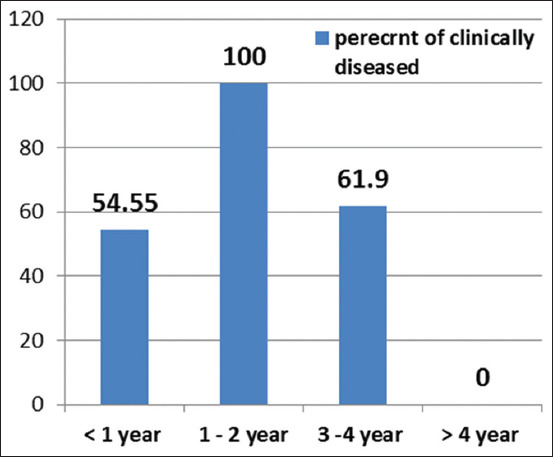
Percent of clinically diseased cattle in relation to the age.

**Figure-2:**
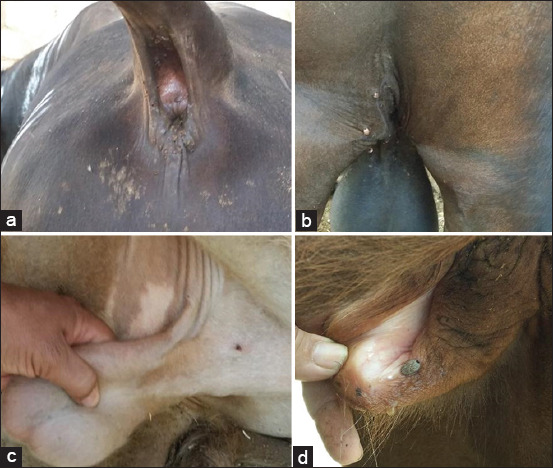
Showing tick infestation on the animal body (a-d) and pale mucus membrane (c and d).

The clinical examination revealed 21 of 40 animals (52.5%) at Education Farm suffered a variety of clinical manifestations, including fever, pale to icteric mucus membranes, hemoglobinuria, and anorexia. All animals were infested with ticks (Table-1) and Figures-1 and 2. The age most severely affected was 1-2 years. Two bulls were clinically diseased. Further, 61.9% of cattle in the 3-4 years age group were clinically diseased, but cattle over 4 years of age were clinically normal cattle. About 54.55% of animals <1-year-old were clinically affected.

**Table-1:** Number and percent of clinically diseased animals according to age and their clinical manifestation.

Age (year)	Total		Clinically diseased	Percent	Clinical signs

	No.	Sex			
<1	11	♂	6	54.55	Icteric mucus membrane, emaciation
1-2	2	♂	2	100	Fever, pale mucus membrane, anorexia
3-4	21	♀	13	61.90	Fever, pale mucus membrane, anorexia, emaciation, hemoglobinuria
>4	6	♀	0	0	Clinically normal cattle
Total	40	-	21	52.5	

### Blood film examination

**Figure-3:**
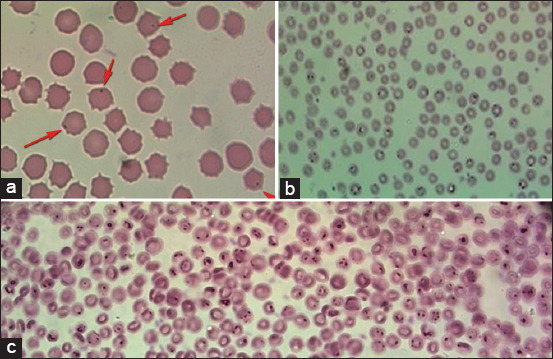
Thin blood films stained with Giemsa stain: (a) RBCs infected with *Anaplasma spp*. (b and c) showing RBCs infected with *Babesia* spp.

Examination of 31 thin blood films showed that 19 cattle suffered from *A. marginale* which appear as dense, homogeneously stained blue-purple inclusions. These inclusions are usually located toward the margins of infected erythrocytes (Figure-3a). In addition, 21 animals showed the presence of *Babesia* spp. These small parasites appear as pairs at an obtuse angle to each other (Figure-3). The severity of infection ranged from mild to severe with mixed infection in some animals. No infections were observed in the ten clinically normal animals.

### PCR assay

**Figure-4:**
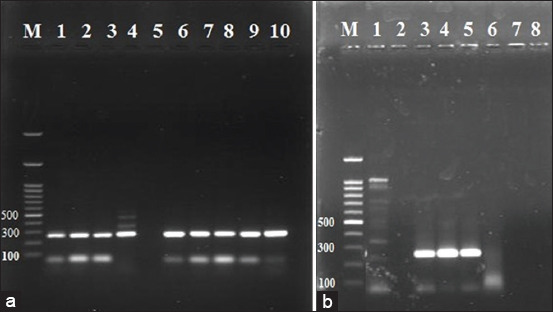
Gel electrophoresis of PCR products of *Anaplasma marginals*, lane 1, 2, 3, 4, 6, 7, 8, 9, 10 in figure (a) are positive on bp 265 in female cows samples, and in figure (b) lane 3, 4, 5 are positive while lane 1 and 6 are negative, the amplified products prepared from positive blood samples except for two samples from calves were negative for *Anaplasma marginale*.

**Figure-5:**
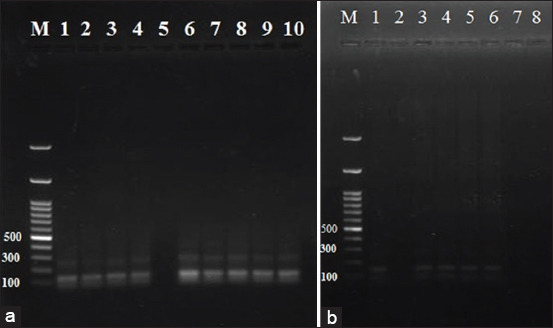
Gel electrophoresis of PCR products of *Baabesia bovis*, lane 1, 2, 3, 4, 6, 7, 8, 9, 10 in figure (a) are positive on bp 166 in cows samples while in figure (b) lane 1, 3, 4, 5, 6 are positive, the amplified products prepared from positive blood samples.

The PCR used DNA from 14 samples, nine from female cattle and five from male calves. Nine samples from cows were positive for *Babesia* and *Anaplasma* by blood film examination. Five calves, three with mixed infection and two calves with *Babesia* were also identified form blood film examination. PCR results show the selected 14 samples as positive for infection with *B. bovis*. *Anaplasma marginale* infection was detected in nine samples of cattle and three samples of calves. The remaining two samples were negative (Figures-4 and 5).

### Biochemical investigation

The biochemical profiles show significantly elevated serum ALT, AST, total bilirubin (T. Bil), and urea in blood parasite-infected female cattle and male calves as compared with the control animals. Significantly elevated TP, creatinine, and globulin serum levels were recorded only in infected female cows, in comparison with the control group (Table-2). Conversely, blood glucose levels were significantly decreased in infected female cattle and male calves. Albumin and A/G ratios were also significantly reduced in infected female cattle in comparison with control animals (Table-2). The oxidative stress profile of infected animals show significant increases in serum MDA and NO; however, both TAC and GSH were significantly reduced (Table-3).

**Table-2:** Some serum biochemical profiles (mean±S.E), for normal and infected cattle.

Biochemical parameters	Cows (female)		Calves (male)	
	
	Control group	Infected group	Control group	Infected group
ALT (U/L)	22.18±1.14	42.03±1.67***	16.20±1.47	37.12±0.83***
AST (U/L)	60.68±1.54	118.88±1.66***	72.44±4.44	119.26±7.75**
T.P (g/dL)	6.56±0.15	7.92±0.26**	6.40±0.20	5.62±0.17*
Albumin (g/dL)	3.42±0.04	3.20±0.03**	3.42±0.18	3.02±0.12
Globulin (g/dL)	3.14±0.12	4.72±0.27**	2.98±0.12	2.60±0.11
A/G Ratio	1.09±0.03	0.69±0.05***	1.16±0.08	1.17±0.06
Glucose (mg/dL)	57.00±2.12	31.68±1.39***	88.40±8.64	37.76±5.04**
Urea (mg/dL)	14.54±1.39	33.40±2.58***	12.76±1.89	26.02±0.83***
Creatinine (mg/dL)	0.60±0.02	1.45±0.15**	0.98±0.41	1.03±0.01
T. Bil. (mg/dL)	0.296±0.01	0.575±0.06*	0.22±0.01	0.52±0.04*

Values represent mean±SE. SPSS version 23 (independent-sample t-test). Significance:

* p<0.05,

** p<0.01,

*** p<0.001 comparing to control cattle. AST=Aspartate aminotransferase, ALT=Alanine aminotransferase, TP=Total protein, A/G ratio=Albumin/globulin ratio, SE=Standard error

**Table-3:** Oxidative stress profiles (mean±S.E), for normal and infected cattle.

Oxidative stress parameter	Cows (female)		Calves (male)	
	
	Control group	Infected group	Control group	Infected group
MDA (nmol/mL)	2.73±0.48	7.04±0.89**	2.79±0.27	5.83±0.48***
NO (µmol/L)	2.09±0.08	1.47±0.04***	1.78±0.0	2.14±0.06**
GSH (mg/dL)	2.25±0.07	0.97±0.24**	3.496±0.20	2.386±0.15**
TAC (mM/L)	1.72±0.08	0.87±0.09***	1.83±0.06	1.23±0.07***

Values represent mean±SE. SPSS version 23 (independent-sample t-test). Significance:

* p<0.05,

** p<0.01,

*** p<0.001 comparing to control cattle. MDA=Malondialdehyde, GSH=Reduced glutathione, NO=Nitric oxide, TAC=Total anti-oxidant capacity, SE=Standard error.

## Discussion

Cattle at the educational farm of the Faculty of Agriculture suffered from a variety of clinical manifestations suspected to be caused by blood parasites. This suspicion was confirmed by blood film and PCR. Our clinical examination of 40 animals during study period revealed 21 (52.5%) suffered from a variety of clinical signs including fever, pale to icteric mucus membranes, hemoglobinuria, and anorexia. All animals examined were infested with ticks (Table-1 and Figures-1 and 2). Blood film examination (Figure-3) revealed *Babesia* and *Anaplasma* coinfection in the herd. Coinfection with other tick-transmitted pathogens is expected to complicate diagnosis in cattle; hence, molecular identification of causative agents is a priority. The present findings agree a previous report [8] that shows that *B. bovis* and *A. marginale* are intraerythrocytic pathogens responsible for the most prevalent and expensive tick-borne diseases in cattle worldwide.

Anaplasmosis is a tick-borne disease where bacteria replicate within the epithelial cells of the tick midgut. The disease is endemic in tropical and subtropical areas worldwide and added that anaplasmosis could be misdiagnosed with tick-borne disease caused by *B. bovis and B. bigemina* [15]. These protozoans have a similar geographical distribution and cause anemia in cattle. Further, besides transmission by ticks, *Babasia* spp. and *A. marginale*, can also be transmitted mechanically [16] by biting flies [4] and needles [17].

In the current study, diagnosis of anaplasmosis in the farm reached 52.5%; 21 of 40 animals. This incidence is higher than the incidence reported previously (28%) [18] using a cELISA. *A. marginale* incidence was determined with DNA in sera using molecular techniques in 20.12% of animals [5].

These differences may be due to different sampling times, sampling schemes, and locations. Notably, the samples in this study were taken during locally limited outbreaks from clinical practice, in contrast to sampling based on an independent, statistically based plan. In endemic areas, clinical cases are rarely observed. Often, infections are seen in naïve animals from disease-free areas. Animals persistently infected can be responsible for outbreaks in a naïve herd, when moved to a disease-free area where vectors are present [19].

The results in the present study revealed a significant increase in ALT and AST in infected cattle. These findings coincide previous work that suggested that increased serum ALT and AST in camels with anaplasmosis might indicate hepatic dysfunction [20]. Furthermore, our results are in accord with the report that elevated ALT and AST levels indicate a harmful impact of toxic metabolites of *Babesia* sp. Such metabolites might lead to impairment of hepatocytes with subsequent release of liver enzymes [21]. Alternatively, increased liver enzymes during *Babesia* infection may result from liver damage and lesions caused by the parasite during proliferation in the blood that indirectly results in hepatic dysfunction [22]. Elevated hepatic enzymes might also follow lysis of the RBCs or hyperbilirubinemia during babesiosis. Finally, the increased level of AST and ALT could be a consequence of damaged heart or skeletal muscles, liver tissues, and RBCs. These tissues may contain a large reservoir of enzymes that might be released into blood [23]. Our result is in agreement with previous studies [24-27].

In this study, our data show a significant increase in TP and globulin in infected female cows in comparison with normal controls. These results are consistent with the previous study that indicates that elevated serum globulin level in the *A. marginale* infected animals could be due to activation of a defensive immune response that leads to an increase in circulating immunoglobulin in the serum [28]. In contrast, hypoalbuminemia is also reported that could be due to disruption in liver function that leads to a decrease in albumin synthesis during *Babesia* infection [9].

Our result revealed a significant increase in T. Bil in infected cattle. Hyperbilirubinemia was previously reported in camels infected with *A. marginale* due to excessive destruction of RBCs and the indirect hepatocellular damage [20]. Furthermore, elevated levels of T. Bil were shown to reflect intravascular and extravascular hemolysis [29]. Our results are also consistent with other investigations [26,28].

A significant decrease in blood glucose level was recorded in our study in infected animals. A reduction in blood glucose level might be caused by the parasite utilization of glucose as well as the liver damage in large ruminants infected by *B. bovis* [30]. This observation is also in accord with other reports [22,31]. Moreover, these findings are consistent with reduced blood glucose levels in 90% of *Babesia canis* infected dogs [32]. This reduction may reflect sepsis that leads to anorexia and impaired liver function.

The results showed significantly elevated serum urea and creatinine levels. This finding could reflect indirect renal tissue damage and the existence of globin catabolites released from hemoglobin breakdown by the reticuloendothelial system through erythrophagocytosis [20] accompanied by hypoxia leading to glomerular dysfunction [33] as well as nephropathy and immune-mediated glomerulonephritis [34].

Parasitic infections result in the activation of inflammatory cells, which are crucial for the host defense. The activation of inflammatory cells stimulates and activates several oxidant-generating enzymes, which are regulated by many pro-inflammatory cytokines such as tumor necrosis factor-α (TNF-α), interleukin-1β (IL-1β), interleukin-6 (IL-6) and others [35]. In babesiosis caused by *B. bovis*, the infection involves production of IL-1β, interleukin-12 (IL-12), gamma interferon (IFN-γ) and TNF-α [35,36]. These mediators activate mononuclear phagocytes/macrophages to release reactive nitrogen intermediates where *B. bovis*-infected erythrocytes stimulate the production of inducible nitric oxide synthesis (iNOS) transcription and nitric oxide by the activated macrophages in bovines with subsequent increased oxidative stress to macromolecules and biomembranes, resulting in augmented lipid peroxidation and MDA ­production [36]. In addition,the enhanced production of the inflammatory cytokines produce large amounts of highly toxic molecules, like ROS including hydroxyl radicals, hydrogen peroxide and superoxide anion, as well as reactive nitrogen species, including nitric oxide, which are capable of degrading many biomolecules, including lipids, proteins, carbohydrates and DNA [35]. Our data showed significantly elevated MDA and NO in infected cattle. These results are similar to increase in both MDA and NO in calves infected with *A. marginale* [7], assumed to be related to the severity of *Anaplasma* infection that is directly associated with oxidative stress and loss of antioxidant reserve. Furthermore, our results agree with the previously recorded significant increase in both MDA and NO in cattle with anaplasmosis [37].

Our results revealed significantly reduced GSH and TAC in the infected cattle, which is consistent with the suggestion that [5] the decrease in GSH in cattle with *A. marginale* infection might be due to either elevated activity of glutathione peroxidase that utilizes GSH to reduce peroxides or to its capacity to directly detoxify ROS. In this study, reduced serum TAC in the infected cattle may result from the exhaustion of antioxidant enzymes that act as scavengers for the free-radical throughout the oxidative process that occurs during *A. marginale* infection of cattle [11,38,39].

## Conclusion

This study revealed a higher incidence of infection with (*B. bovis* and *A. marginale*) pathogens of medical and veterinary relevance in Aberdeen Angus cattle in New Valley Oasis, Egypt. Furthermore, mixed infection with *B. bovis* and *A. marginale* is associated with hypoglycemia and renal and hepatic dysfunction and exerts severe oxidative stress on infected animals. Additional studies are required to define clinical impacts, epidemiology, and immune profiles of infected animals.

## Authors’ Contributions

NN, YFE, and HH prepared the original idea. NN and YFE conceptualized the aim, design, and plan of the study and prepared the tables and the figures. NN and HH made blood sampling. RME did all the molecular investigations. YFE wrote the discussion related to the molecular study in the original manuscript draft. NN analyzed the biochemical and oxidative stress parameters, did the statistical analysis and wrote the original manuscript draft. All authors contributed to revising the final draft of the manuscript. All authors read and approved the final manuscript.
